# Socioeconomic and Behavioral Factors Associated with Tuberculosis Diagnostic Delay in Lima, Peru

**DOI:** 10.4269/ajtmh.17-0096

**Published:** 2018-04-23

**Authors:** Lily V. Bonadonna, Matthew J. Saunders, Heinner Guio, Roberto Zegarra, Carlton A. Evans

**Affiliations:** 1 The University of Michigan, Ann Arbor, Michigan;; 2 Instituto Nacional de Salud, Lima, Perú;; 3 Innovación Por la Salud Y Desarrollo (IPSYD), Asociación Benéfica PRISMA, Lima, Perú;; 4 Innovation For Health And Development (IFHAD), Laboratory of Research and Development, Universidad Peruana Cayetano Heredia, Lima, Perú;; 5 Infectious Diseases & Immunity, Imperial College London, and Wellcome Trust Imperial College Centre for Global Health Research, London, United Kingdom

## Abstract

Early detection and diagnosis of tuberculosis (TB) is a global priority. Prolonged symptom duration before TB diagnosis is associated with increased morbidity, mortality, and risk of transmission. We aimed to determine socioeconomic and behavioral factors associated with diagnostic delays among patients with TB. Data were collected from 105 patients with TB using a semi-structured interview guide in Lima, Peru. Factors associated with diagnostic delay were analyzed using negative binomial regression. The median delay from when symptoms commenced and the first positive diagnostic sample in public health facilities was 57 days (interquartile range: 28–126). In multivariable analysis, greater diagnostic delay was independently associated with patient older age, female gender, lower personal income before diagnosis, living with fewer people, and having more visits to professional health facilities before diagnosis (all *P* < 0.05). Patients who first sought care at a private health facility had more visits overall to professional health facilities before diagnosis than those who first sought care from public or insured employee health facilities and had longer diagnostic delay in analysis adjusted for age and gender. Patients with TB were significantly more likely to first self-medicate than to visit professional health facilities before diagnosis (*P* = 0.003). Thus, diagnostic delay was prolonged, greatest among older, low-income women, and varied according to the type of care sought by individuals when their symptoms commenced. These findings suggest that TB case-finding initiatives should target vulnerable groups in informal and private health facilities, where many patients with TB first seek health care.

## BACKGROUND

Tuberculosis (TB) is the most frequent infectious cause of death worldwide, with the highest rates occurring in low- and middle-income countries. Of the 10.4 million people estimated to have developed TB in 2015, only 57% of cases were notified.^1^ Peru has the second highest TB burden in the Americas with an estimated incidence rate of 119 per 100,000 population in 2015.^2^ Despite an acclaimed National TB Program (NTP) that includes directly observed therapy and offers diagnostic tests, treatment, and psychosocial services free of direct charges, the TB burden remains high and cases of resistant TB are increasing.^2^

Early detection, diagnosis, and treatment of TB is important for global control and elimination and is emphasized in the World Health Organization’s (WHO) End TB Strategy.^3,4^ Delays in diagnosis and low case detection can lead to prolonged periods of infectiousness and transmission, and increased risk of adverse treatment outcomes, including death.^5^ Previous studies in Peru have reported long delays in diagnosis that contribute to TB-related morbidity and mortality.^6,7^ To identify people with undiagnosed TB and reduce detrimental diagnostic delays, case-finding initiatives must be developed to more effectively target vulnerable groups and settings.^1^

The reasons and risk factors for diagnostic delay have been reported to vary in different parts of the world.^8–10^ Previous studies have reported that patients’ health-seeking behaviors are influenced by numerous factors, including socioeconomic status, stigma, perceptions of illness severity, symptom recognition, time taken to reach health services, and perceived quality and costs of health services.^8,11^ In Peru, the NTP regulates TB treatment in public health posts, public hospitals, and insured employee clinics. The NTP recommends that individuals seek free TB testing after 15 consecutive days of cough productive of sputum and predominantly relies on passive case finding to diagnose new TB cases. In addition, contacts of known patients with TB are an established high risk group for which Peruvian policy recommends active case finding.^12,13^

In this study, we aimed to investigate health-seeking behaviors and determine the socioeconomic and behavioral factors associated with diagnostic delay among patients with TB in Lima and Callao, Peru.

## METHODS

### Study design, setting, and participants.

**Figure 1.**
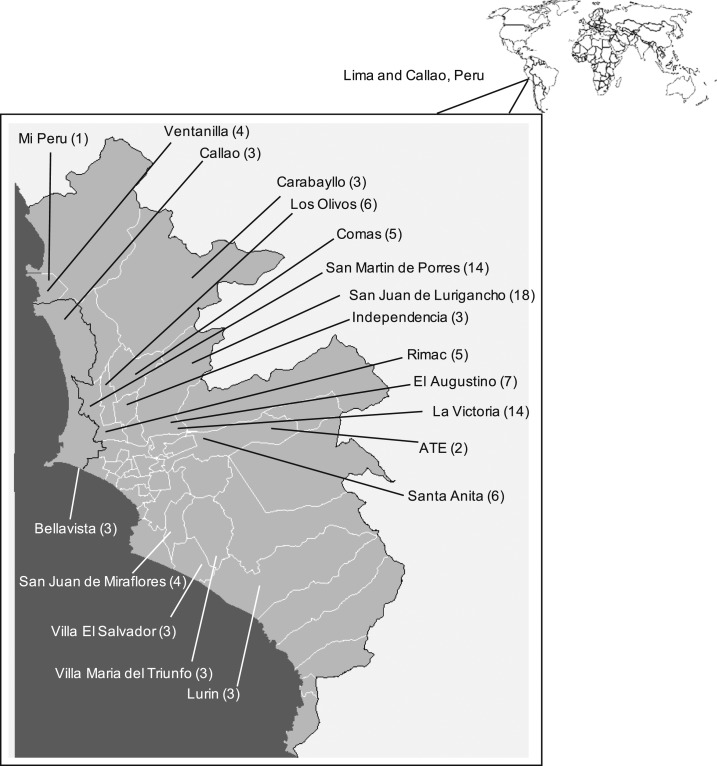
All visited city districts. (The number of persons diagnosed with tuberculosis who were interviewed in each city district are shown in parentheses.)

In 2015, we performed a cross-sectional, observational study of patients with TB in Lima and Callao, Peru. These are the most densely populated areas of the country, comprising 50 city districts housing an estimated 9.8 million people. We aimed to collect data in districts with the highest TB notification rates and relied on data provided to us by the regional coordinators of the NTP to make this selection. Although this was the best available source of data, it may contain inaccuracies because the NTP largely relies on paper monitoring systems for data tracking. We chose 19 districts in geographically distinct zones and visited public health posts with the highest respective TB notification rates within these zones (Figure 1). Study field sites were diverse, varying from desert shantytown communities to developed, inner-city centers. All health posts had a designated TB treatment program that was regulated under the jurisdiction of the NTP.

Participants were eligible to be invited to join the study if they were aged ≥ 18 years and were presently taking treatment for their first episode of sputum smear microscopy-positive, pulmonary TB, irrespective of drug resistance. We selected participants by convenience sampling whereby we approached eligible participants who were taking TB treatment at a selected health post at the time of our visit. To ensure a balanced sample from the study setting, we aimed to recruit an approximately equal number of participants from each health post. All individuals provided written informed consent before participating. The study received ethical approval from the Peruvian National Institutes of Health and the University of Michigan, USA.

### Procedures.

A semi-structured interview guide was used to explore the following themes: sociodemographics; household environment and family composition; past medical history; history of TB symptoms; and health-seeking behaviors. This guide was developed from a prior WHO survey of diagnostic and treatment delay, piloted, and modified based on responses.^8^ Medical charts were also reviewed. All interviews were conducted in Spanish by one individual (L. V. B.) trained in data collection methods. Interviews took place at the public health post or in the participant’s home, depending on the patient’s preference, in one or two meetings lasting 30 minutes to 1 hour.

### Measures.

Diagnostic delay was defined as the number of days between self-reported symptom onset and the first sputum smear microscopy-positive TB sample recorded in a public health post. Patients were asked to describe the symptoms they considered to be associated with their TB diagnosis before we specifically asked them if they had experienced cough, fever, weight loss, and/or hemoptysis. We reduced risks of reporting and recall biases by questioning patients and reconfirming their original responses.

Socioeconomic factors measured included: gender, age, civil status, educational background (level of schooling completed), number of cohabitants, whether the patient lived with a person who previously or presently had TB, income level before symptom onset, and history of alcohol and drug use.

We recorded patients’ histories of health-seeking behavior. We categorized professional health facilities attended into one of: 1) public health post; 2) public hospital; 3) insured employee clinic; or 4) private clinic. We defined health-seeking activity in pharmacies or taking natural medicines as self-medication if participants were not directed to buy medications by a physician.

### Statistical analysis.

All analyses were performed using Stata (version 13, StataCorp, College Station, TX). All *P* values generated were two sided with significance assessed at the 5% level. Continuous data were summarized by medians and interquartile ranges (IQR). A Mann–Whitney test was used to compare the overall number of visits to professional health facilities among people whose first contact with professional health facilities was to consult private clinics versus those who first visited public or insurer clinics. Categorical data were summarized by proportions and compared using the proportions test with their 95% confidence intervals (95% CI).

Negative binomial regression was used to investigate sociodemographic and behavioral factors associated with diagnostic delay because these count data were not normally distributed and were over-dispersed. All variables were first examined in unadjusted univariable analysis with box plots plotted and rate ratios (RR) with 95% CI and *P* values calculated. For the purposes of this analysis, we dichotomized patient income defining patients as having a lower income if they earned less than or equal to the median value. We also dichotomized education level if patients had completed secondary education or not. Age, number of cohabitants, and number of visits to professional health facilities before diagnosis were analyzed in univariable analysis both as continuous variables, and as variables dichotomized by the median. We chose not to analyze the association of tobacco, alcohol, and drug use with diagnostic delay because the number of patients who reported current use was negligible. All variables were subsequently individually examined in analysis adjusted for age and gender. Finally, a multivariable model was built to calculate adjusted rate ratios (aRR) with standard errors adjusted for clustering by district of recruitment. In this analysis, we initially included all variables that were plausibly associated with diagnostic delay and showed some evidence of association in univariable analysis. Age, number of cohabitants, and number of visits to professional health facilities before diagnosis were considered as continuous variables. The maximal multivariable model was reduced by eliminating variables sequentially and comparing log-likelihood between models. Interactions were tested between variables as detailed in Table 2 by including interaction parameters and comparing models using likelihood ratio tests. We further investigated the associations between variables to determine collinearity. To confirm that the negative binomial model was an appropriate analysis strategy, we calculated the over-dispersion parameter and performed a likelihood test comparing the negative binomial model to a Poisson model. To assess goodness of fit, we calculated the scaled Pearson’s χ^2^ and scaled deviance statistics. Finally, we performed a sensitivity analysis excluding one outlying patient who reported 33 months of diagnostic delay.

To estimate the relative contribution of risk factors to diagnostic delay, population attributable fractions (PAFs) were estimated from the final regression model using the “*punaf*” Stata command. This method estimates and compares the logs of two scenario means, the observed data scenario, and a theoretical scenario in which exposure variables are set to specific values. For binary variables (sex and income), the theoretical scenario was to remove the risk factor from the population. For age and number of cohabitants, the theoretical scenarios were set as the median values (28 years and four cohabitants, respectively). For number of visits to professional health services before diagnosis, the theoretical scenario was set as one visit.

## RESULTS

### Baseline data.

We recruited 105 patients with TB during the study period. The median age was 28 years (IQR: 22–49), 40/105 (38%) were female, and 82/105 (78%) had completed secondary education. Patients personally earned a medium monthly income of 218 (IQR: 116–349) United States dollars (USD). The legal minimum wage in Peru was 215 USD monthly. Baseline characteristics of all participants are detailed in Table 1.

**Table 1:** Baseline characteristics of the study population

	Variable	Units	Patients with TB (*N* = 105)
General	Age	median (IQR)	28 (22–49)
	Female gender	*n* (%)	40 (38)
	Monthly personal income	median USD (IQR)	218 (116–349)
Civil status	Single	*n* (%)	73 (70)
	Married	*n* (%)	13 (12)
	Lived with partner	*n* (%)	16 (15)
	Separated	*n* (%)	1 (1)
	Widow	*n* (%)	2 (2)
Education	Complete primary education	*n* (%)	98 (93)
	Complete secondary education	*n* (%)	82 (78)
	Postsecondary technical degree	*n* (%)	11 (11)
	Postsecondary university degree	*n* (%)	5 (5)
Tobacco use	Current	*n* (%)	0 (0)
	Occasionally used before diagnosis	*n* (%)	20 (19)
	Excessively used before diagnosis (everyday)	*n* (%)	41 (39)
Alcohol use	Current	*n* (%)	1 (1)
	Occasionally used before diagnosis	*n* (%)	17 (16)
	Excessively used before diagnosis (everyday)	*n* (%)	4 (4)
Drug use	Current	*n* (%)	1 (1)
	Occasionally used before diagnosis	*n* (%)	17 (16)
	Excessively used before diagnosis (everyday)	*n* (%)	4 (4)
Household characteristics	Number of cohabitants	median; IQR	4 (2–5)
	Number of economically contributing cohabitants	median; IQR	1 (1–2)
	Lived with a person who previously had TB	*n* (%)	25 (24)
	Lived with a person who presently had TB	*n* (%)	9 (9)
	Number or rooms in the household	*n* (%)	4 (2–6)
	Shared a bedroom	*n* (%)	49 (47)
	Shared a bed	*n* (%)	32 (30)
Behaviors	First health behavior was to consult professional health facilities rather than self-medicating	*n* (%)	37 (35)
	First contact with professional health facilities was to consult a private clinic rather than a public or employer-insured health facility	*n* (%)	43 (41)
	Number of visits to professional health facilities before diagnosis	median; IQR	2 (2–3)

IQR = interquartile range; TB = tuberculosis.

### Diagnostic delay.

**Figure 2.**
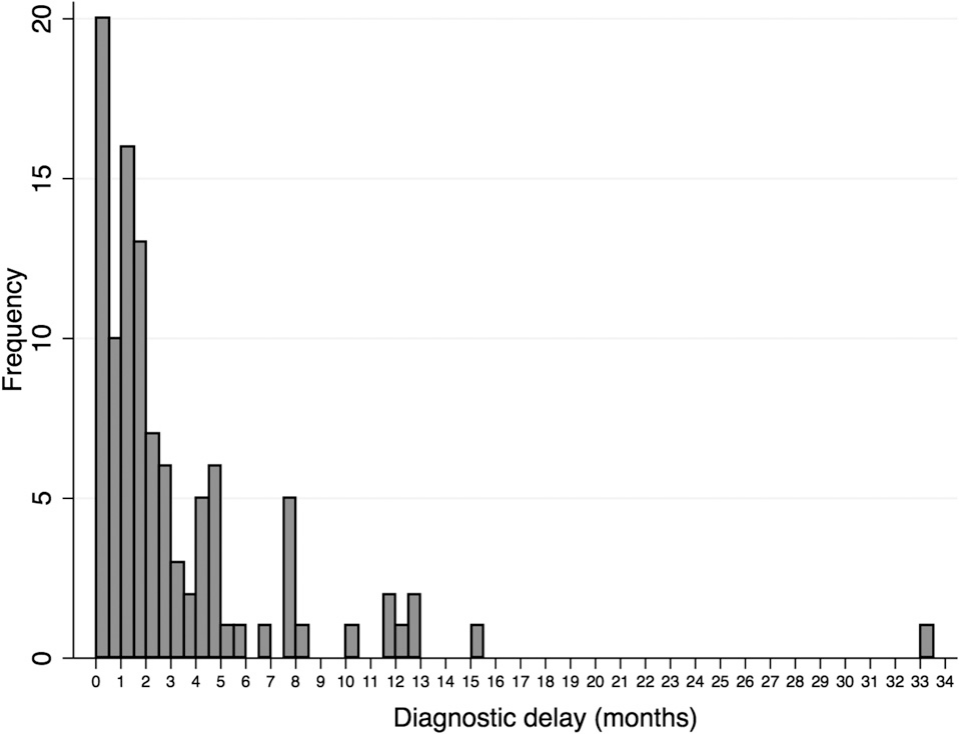
Histogram of diagnostic delay. Diagnostic delay is defined as the number of months from tuberculosis symptom onset until the first clinic visit that led to a sputum smear microscopy-positive sample from the National TB Program in a public health facility.

The median diagnostic delay was 57 days (IQR: 28–126). Twenty percent of patients were diagnosed within 15 days of their reported first identification of symptoms, 27% within 1 month, 52% within 2 months, and 86% within 6 months. The distribution of delay was skewed and over-dispersed (Figure 2).

### Sociodemographic factors.

**Figure 3.**
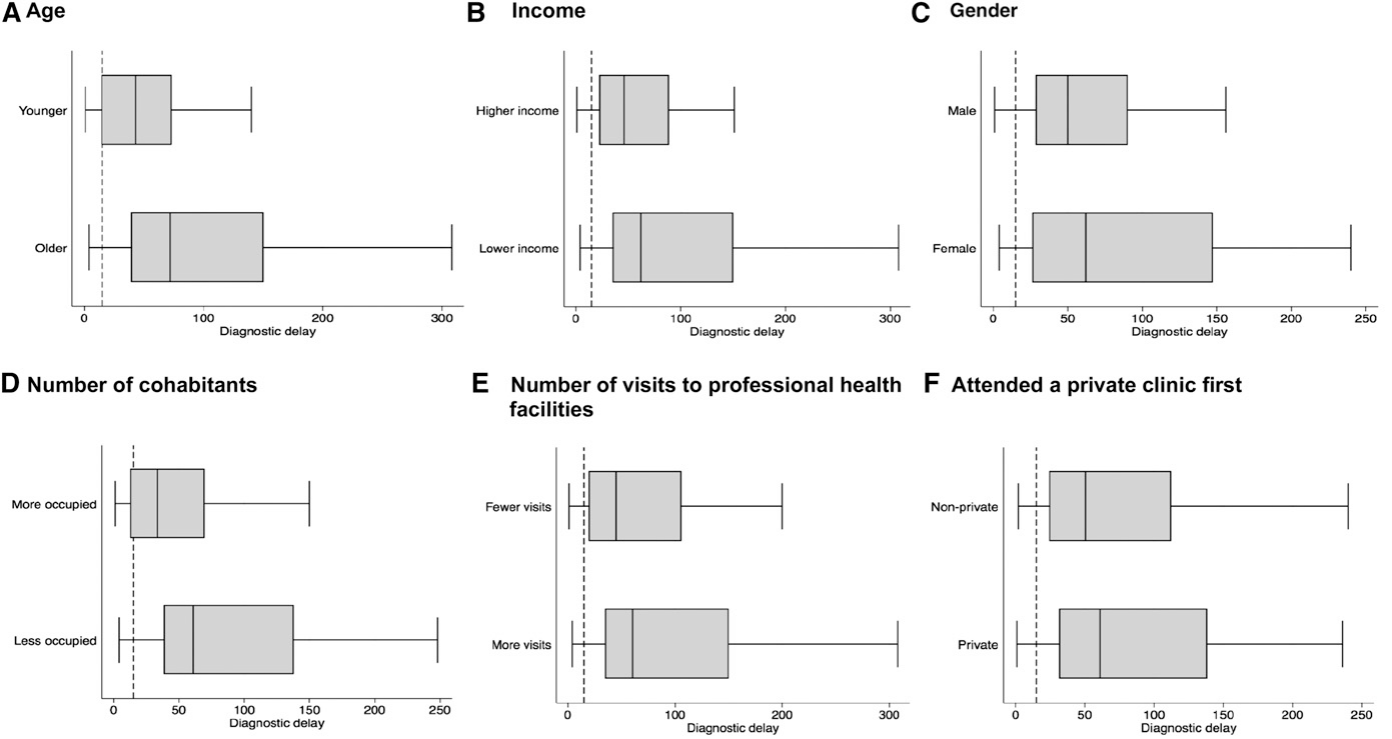
Box plots of factors associated with diagnostic delay, which is indicated in days. Note: for the purposes of the figure, continuous variables (age, income, number of cohabitants, and number of visits to professional health services) were dichotomized according to the median values (28; $218; four cohabitants and two visits, respectively). The dotted vertical reference line indicates 15 days. The Peruvian National TB Program recommends that all people with a cough lasting for 15 days should attend health services to receive a free sputum test for tuberculosis.

Table 2 presents factors associated with diagnostic delay in univariable and multivariable analyses. In univariable analysis, longer diagnostic delay was associated with older age (RR for each additional integer of age = 1.01; 95% CI: 1.00–1.03, *P* = 0.007), female gender (RR = 1.6; 95% CI: 1.0–2.4, *P* = 0.03), lower income (RR = 1.7; 95% CI: 1.1–2.5, *P* = 0.01), and number of cohabitants (RR for each additional cohabitant = 0.88; 95% CI: 0.81–0.96, *P* = 0.004). In the multivariable model adjusted for clustering by district, longer diagnostic delay remained significantly associated with older age (aRR = 1.01; 95% CI: 1.00–1.03, *P* = 0.01), female sex (aRR = 1.5; 95% CI: 1.1–2.2, *P* = 0.02), lower income (aRR = 1.5; 95% CI: 1.1–2.1, *P* = 0.03), and number of cohabitants (aRR = 0.87; 95% CI: 0.81–0.94, *P* < 0.001). Figure 3 demonstrates the associations between these factors and diagnostic delay.

**Table 2:** Factors associated with tuberculosis (TB) diagnostic delay

		Median diagnostic delay (IQR)	Univariable analysis		Analysis adjusted for age and sex		Multivariable analysis including adjustment for district			
			RR (95% CI)	*P* value	aRR (95% CI)	*P* value	aRR (95% CI)	*P* value	aPAF (95% CI)	*P* value
Socioeconomic factors										
Age (continuous)		*	1.01 (1.00–1.03)	0.007	*	*	1.01 (1.00–1.03)	0.01	12 (1.0–22)	0.05
Age group	18–27	43 (15–73)	Ref	Ref	*	*	*	*	*	*
	≥ 28	72 (40–150)	2.0 (1.3–2.9)	0.001	*	*	*	*	*	*
Gender	Male	50 (29–90)	Ref	Ref	*	*	Ref	Ref	Ref	Ref
	Female	62 (27–147)	1.6 (1.0–2.4)	0.03	*	*	1.5 (1.1–2.2)	0.02	16 (1.2–30)	0.04
Education	Completed secondary	51 (28–104)	Ref	Ref	Ref	Ref	–	–	–	–
	Not completed secondary	63 (28–175)	1.3 (0.79–2.1)	0.3	0.91 (0.56–1.6)	0.8	–	–	–	–
Income	Higher	46 (23–89)	Ref	Ref	Ref	Ref	Ref	Ref	*	*
	Lower	62 (36–150)	1.7 (1.1–2.5)	0.01	1.5 (1.0–2.2)	0.05	1.5 (1.1–2.1)	0.03	20 (1.4–36)	0.04
Number of cohabitants (continuous)		*	0.88 (0.81–0.96)	0.004	0.87 (0.80–0.95)	0.007	0.87 (0.81–0.94)	< 0.001	7.0 (1.5–12)	0.01
Number of cohabitants	More (> 4)	34 (13–70)	Ref	Ref	Ref	Ref	*	*	*	*
	Less (≤ 4)	61 (39–138)	2.0 (1.3–3.1)	0.001	2.0 (1.4–3.0)	0.001	*	*	*	*
Lived with a person who had previously had TB	No	59 (29–126)	Ref	Ref	Ref	Ref	–	–	–	–
	Yes	51 (28–140)	0.84 (0.52–1.4)	0.5	0.93 (0.59–1.5)	0.8	–	–	–	–
Lived with a person who presently had TB	No	55 (29–126)	Ref	Ref	Ref	Ref	–	–	–	–
	Yes	61 (26–150)	1.1 (0.53–2.3)	0.8	0.97 (0.48–2.0)	0.9	–	–	–	–
Number of rooms in the household (continuous)		*	0.96 (0.88–1.0)	0.7	0.99 (0.91–1.1)	0.8	–	–	–	–
Shared a bedroom	No	57 (28–138)	Ref	Ref	Ref	Ref	–	–	–	–
	Yes	55 (28–126)	0.75 (0.50–1.1)	0.3	0.95 (0.63–1.4)	0.8	–	–	–	–
Shared a bed	No	60 (17–113)	Ref	Ref	Ref	Ref	–	–	–	–
	Yes	54 (30–135)	0.77 (0.50–1.2)	0.2	1.0 (0.65–1.6)	0.9	–	–	–	–
Behavioral factors										
First health behavior was to self-medicate	No	61 (35–156)	Ref	Ref	Ref	Ref	–	–	–	–
	Yes	52 (27–117)	0.83 (0.54–1.3)	0.4	0.83 (0.56–1.2)	0.4	–	–	–	–
First contact with professional health facilities was to consult a private clinic	No	51 (25–112)	Ref	Ref	Ref	Ref	–	–	–	–
	Yes	61 (32–138)	1.4 (0.94–2.1)	0.1	1.5 (1.0–2.2)	0.05	–	–	–	–
Number of visits to professional health facilities before diagnosis (continuous)		*	1.4 (1.1–1.7)	0.001	1.3 (1.1–1.6)	0.005	1.4 (1.1–1.7)	0.005	37 (10–56)	0.01
Number of visits to professional health facilities before diagnosis	Fewer ≤ 2	45 (20–106)	Ref	Ref	Ref	Ref	*	*	*	*
	More > 2	61 (35–150)	1.8 (1.2–2.6)	0.006	1.6 (1.1–2.3)	0.02	*	*	*	*

aPAF = adjusted population attributable fraction; aRR = adjusted rate ratio; 95% CI = 95% confidence interval; IQR = interquartile range; RR = rate ratio. Ref indicates the reference group used for the regression analysis. Asterisks indicate data that were removed from the regression model (see Methods section). Interactions in the multivariable model tested between age and sex; age and income; sex and income; age and number of cohabitants; sex and number of cohabitants; income and number of cohabitants were not statistically significant.

### Health-seeking behaviors.

**Figure 4.**
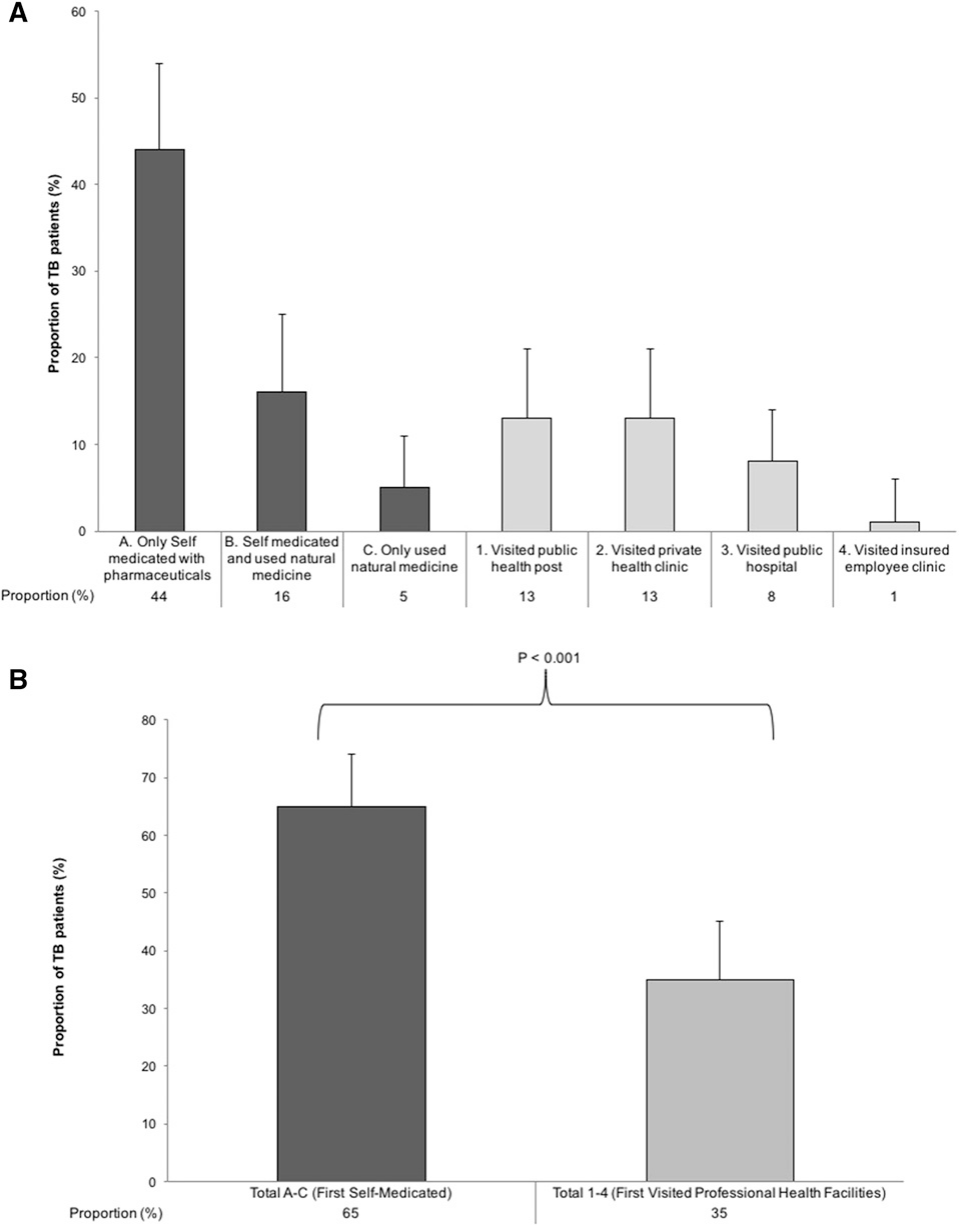
First health-seeking behaviors. Error bars represent 95% confidence intervals. (**A**) The proportion of patients with tuberculosis analyzed by their first health behavior. (**B**) The *P* value represents a two-sample proportion test comparing the proportion of people who self-medicated vs. the proportion of people who visited professional health facilities.

As reported by 42 (44%) patients, the most common initial health-seeking behavior was to self-medicate with pharmaceuticals bought without a physician’s prescription (Figure 4A). Patients were significantly more likely to first self-medicate with pharmaceuticals and/or natural medicines than visit professional health facilities (*P* < 0.001) (Figure 4B). However, these patients who first self-medicated with pharmaceuticals and/or natural medicines had no increased diagnostic delay compared with patients who first sought care at a professional health facility (RR = 0.83; 95% CI: 0.54–1.3, *P* = 0.4).

**Figure 5.**
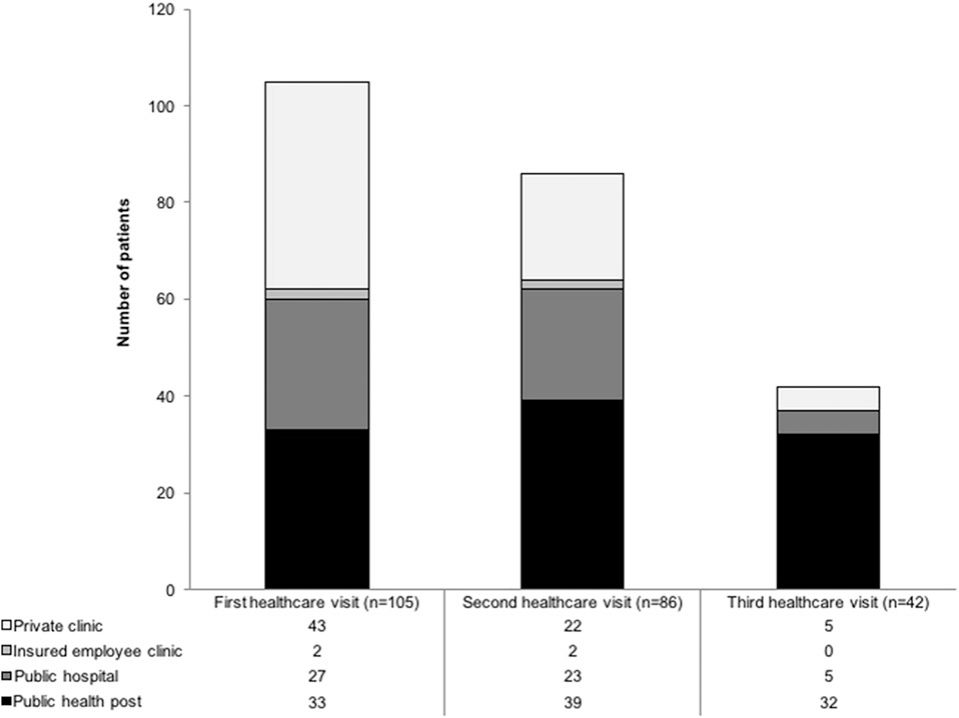
Number of patients with tuberculosis visiting various professional health institutions.

All patients eventually did visit professional health facilities and after this, 86 (82%) went on to seek care from a second professional health facility; and 42 (40%) from a third. The distribution of facilities from which patients sought care on their first, second, and third attempt is shown in Figure 5. For their first visit to a professional health facility, 41 (43%) patients sought care from a private clinic. The median number of visits to professional health facilities before diagnosis and subsequent treatment was 2 (IQR: 2–3). Patients who had more visits to professional health facilities before diagnosis had increased diagnostic delay in multivariable analysis (aRR for each additional visit = 1.4; 95% CI: 1.1–1.7, *P* = 0.005) (Table 2). First seeking care at a private clinic tended to be associated with increased diagnostic delay in univariable analysis and analysis adjusted for age and sex, but not in multivariable analysis (Table 2). These patients who first sought care at private clinics had more visits to professional health facilities before diagnosis than those who sought care from public or insurer-based facilities on their first attempt (median 3 versus 2, respectively; Mann–Whitney test: *P* = 0.02).

### Multivariable regression.

We did not identify any significant interactions between variables, as detailed in Table 2. For variables included in the final model, the only association we identified was between sex and low income, with females more likely to have a low income than males (68% versus 40%, *P* = 0.006). However, both of these variables were independently associated with diagnostic delay in our final model and did not interact. The over-dispersion parameter for the final model was 0.84, and the likelihood ratio test comparing the negative binomial model to a Poisson model indicated that a negative binomial model was appropriate (*P* < 0.001). The scaled Pearson’s χ^2^ and deviance statistics were 1.20 and 1.19, respectively, indicating a reasonable goodness of fit. In the sensitivity analysis excluding the one outlying observation, the results showed only minor, nonsignificant changes (data not shown).

### PAF.

The adjusted PAF of diagnostic delay explained by exposure variables are shown in Table 2. If all patients with TB had been diagnosed on their first visit to professional health services, this analysis predicted that diagnostic delay would have been reduced by 37%.

## DISCUSSION

Early detection and diagnosis of TB are important components of global TB control and elimination efforts. Delays in diagnosis lead to prolonged periods of infectiousness and therefore increase community disease burden.^14^ In this cross-sectional study, we found that patients in Lima and Callao, Peru, experienced symptoms of TB for approximately 2 months before receiving a diagnosis that ultimately resulted in treatment initiation. This diagnostic delay is similar to that seen in other studies in Peru^6,15^ and comparable to delays reported from Malaysia, South Africa, Korea, and Botswana.^16^ To reduce the detrimental consequences associated with diagnostic delay, case-finding initiatives may shift focus from passive strategies that rely on ill persons to independently seek testing for TB.^17^ Instead, active case-finding initiatives could target particularly vulnerable individuals in locations where they are most likely to first seek health care.^18^ In this study, we identified specific socioeconomic and behavioral factors that have important implications for how these case-finding initiatives may be designed and implemented.

In our study, lower personal income before diagnosis was associated with increased diagnostic delay. This may be due to lack of funds to attend professional health facilities, lack of time, or other poverty-related factors.^19^ TB disproportionately affects low-income populations, and the direct and indirect costs associated with TB diagnosis and treatment hamper access to health care, contribute to further impoverishment, and are associated with adverse TB treatment outcomes, including death and loss to follow-up.^20–23^ For example, a previous study in Lima found that the perception of long waiting times deterred individuals from attending public health facilities.^24^ The present study supports these previous findings and highlights the necessity of reducing the financial and time constraints associated with receiving a TB diagnosis.

Although TB-testing is free of direct charges in public health facilities, we found that many patients first visited private clinics, an observation previously reported by other studies.^25,26^ This could be due to perceived increased quality of care or perceived decreased waiting time. We did not record if patients had a positive diagnostic test in a private clinic before subsequently visiting a public health facility to initiate TB treatment free of direct charges. Patients’ first preference for private clinics is particularly concerning in our setting; however, because these patients ultimately made more visits to professional health facilities and tended to have longer delay before receiving a diagnosis that resulted in TB treatment initiation. The association observed between care at a private clinic and diagnostic delay may not have been statistically significant in multivariable analysis because it was likely mediated by an overall increased number of visits to professional health facilities before diagnosis. These findings suggest that it is important that public health posts and insured employee clinics gain community trust and recognition as viable facilities to seek health care.

In this study, the great majority of patients with TB visited at least two health facilities before diagnosis. We did not characterize what tests and treatment, including TB tests, patients received at each visit and were unable to quantitatively determine when in the diagnostic delay time span these visits occurred. However, our results suggest that if all patients with TB had been diagnosed on their first visit to a professional health facility, diagnostic delay would have been reduced by 37%. These findings highlight the importance of collaboration between the NTP and private professionals; health professional education to promote TB as a possible diagnosis; and advising patients to seek free diagnosis and treatment in public health facilities. Our finding that approximately one in five patients with symptoms of TB received a TB diagnosis on their first visit to health facilities supports previous research characterizing a “know-do” gap among health professionals: although recommended TB testing and treatment strategies are known and implemented in theory, health professionals typically choose other practices to investigate and manage patients presenting with symptoms of TB.^27^

When patients began to experience TB symptoms, they reported that self-medicating with pharmaceuticals was the most common initial health-seeking behavior. This may be due to the speed and/or perceived lower cost of treatment in pharmacies or with natural medicines. In other settings, self-medicating has been associated with increased diagnostic delay.^28–31^ In our study, however, we did not demonstrate a significant difference in diagnostic delay between people who first sought symptom-alleviating medications from pharmacies and those who first consulted professional health facilities. In Peru, similar interactions between patients and the traditional healers they visit before seeking biomedical care have been reported.^14^ Other studies have found poor referral rates from pharmacies to public health facilities.^16,26,32^ Future active case-finding initiatives could actively target pharmacies because of the high number of symptomatic patients who buy medications before attending professional health facilities to receive a diagnostic test. These type of initiatives, however, should be designed to reduce stigmatization of people and communities.

We found that patients who lived with fewer people had longer diagnostic delay. In this setting, living with more cohabitants may be indicative of greater potential to receive social support. These data support similar qualitative results from the current setting^33^ and the Philippines, describing strong social support as a major contributor to seeking care from professional health facilities.^25^ In addition, our findings support another study from Uganda that describes health-seeking behavior as part of a social process whereby recommendations, financial assistance, and guidance from contacts strongly influence decision-making.^34^

In the present study, diagnostic delay was longer in women than it was in men. This supports previous qualitative work that describes unique obstacles to women’s health care–seeking behavior and a commonly held perception of women’s TB care as secondary to men in our setting.^35^ Previous quantitative research in Lima and other resource-constrained countries have also reported women experiencing longer diagnostic delay than men.^15,36,37^ Diagnostic delays were also significantly longer in older participants, supporting previous work in Peru.^6^ Thus, to maximize their effectiveness, case-finding initiatives should be designed as inclusive and sensitive to the specific health care needs of these populations, which should be further investigated qualitatively.

Limitations to our study include recall bias because participants provided responses retrospectively, after they had been admitted to a TB treatment program. We aimed to reduce this bias by questioning and reconfirming participants’ responses. In addition, selection bias may have occurred as we used convenience sampling to recruit patients taking treatment in public health posts and were unable to recruit patients who never initiated treatment, or who had TB but were never diagnosed. We recruited patients from health posts in diverse districts of the city with the highest TB burden and do not believe that these biases alone could explain our results. Another general criticism of cross-sectional analysis is the difficulty of clearly defining causative links between factors studied and the outcome of interest, defined as diagnostic delay in our study. However, the factors we identified have plausible associations with diagnostic delay and have been shown to be reproducible across a variety of settings. Furthermore, we did not characterize other variables that may have contributed to diagnostic delay, such as travel time to health facilities, and our relatively small sample size may have limited the power of the study to detect small differences in diagnostic delay. Finally, after the diagnostic delay that we studied, we did not investigate subsequent treatment delay, which describes the time after diagnosis by a health professional until the start of treatment. Treatment delay and other factors, including previous, erroneous diagnoses received by patients before their TB diagnosis, should be further investigated to characterize other barriers to receiving a TB diagnosis within the health system.

In conclusion, diagnostic delay is common, prolonged, and influenced by several important socioeconomic and behavioral factors. Our findings have important implications for policymakers and researchers to facilitate the design and implementation of active case-finding initiatives.^17^
